# Model-Free Machine Learning in Biomedicine: Feasibility Study in Type 1 Diabetes

**DOI:** 10.1371/journal.pone.0158722

**Published:** 2016-07-21

**Authors:** Elena Daskalaki, Peter Diem, Stavroula G. Mougiakakou

**Affiliations:** 1 Diabetes Technology Research Group, ARTORG Center for Biomedical Engineering Research, University of Bern, Murtenstrasse 50, 3008 Bern, Switzerland; 2 Division of Endocrinology, Diabetes and Clinical Nutrition, Bern University Hospital “Inselspital”, 3010 Bern, Switzerland; University of Bremen, GERMANY

## Abstract

Although reinforcement learning (RL) is suitable for highly uncertain systems, the applicability of this class of algorithms to medical treatment may be limited by the patient variability which dictates individualised tuning for their usually multiple algorithmic parameters. This study explores the feasibility of RL in the framework of artificial pancreas development for type 1 diabetes (T1D). In this approach, an Actor-Critic (AC) learning algorithm is designed and developed for the optimisation of insulin infusion for personalised glucose regulation. AC optimises the daily basal insulin rate and insulin:carbohydrate ratio for each patient, on the basis of his/her measured glucose profile. Automatic, personalised tuning of AC is based on the estimation of information transfer (IT) from insulin to glucose signals. Insulin-to-glucose IT is linked to patient-specific characteristics related to total daily insulin needs and insulin sensitivity (SI). The AC algorithm is evaluated using an FDA-accepted T1D simulator on a large patient database under a complex meal protocol, meal uncertainty and diurnal SI variation. The results showed that 95.66% of time was spent in normoglycaemia in the presence of meal uncertainty and 93.02% when meal uncertainty and SI variation were simultaneously considered. The time spent in hypoglycaemia was 0.27% in both cases. The novel tuning method reduced the risk of severe hypoglycaemia, especially in patients with low SI.

## Introduction

Type 1 diabetes (T1D) is a metabolic disease characterised by uncontrolled blood glucose levels, due to the absence or malfunction of insulin. The Artificial Pancreas (AP) system aims to simulate the function of the physiological pancreas and serve as an external automatic glucose regulation system. AP combines a continuous glucose monitor (CGM), a continuous subcutaneous insulin infusion (CSII) pump and a control algorithm which closes the loop between the two devices and optimises the insulin infusion rate.

An important challenge in the design of efficient control algorithms for AP is the use of the subcutaneous route both for glucose measurement and insulin infusion (sc-sc route); this introduces delays of up to 30 minutes for sc glucose measurement and up to 20 minutes for insulin absorption. Thus, a total delay of almost one hour restricts both monitoring and intervention in real time. Moreover, glucose is affected by multiple factors, which may be genetic, lifestyle and environmental. With the improvement in sensor technology, more information can be provided to the control algorithm (e.g. more accurate glucose readings and physical activity levels); however, the level of uncertainty remains very high. Last but not least, one of the most important challenges emerges from the high inter- and intra-patient variability, which dictate personalised insulin treatment.

Along with hardware improvements, the challenges of the AP are gradually being addressed with the development of advanced algorithmic strategies; the strategies most investigated clinically are the Proportional Integral Derivative (PID) [1], the Model Predictive Controller (MPC) [2]-[7] and fuzzy logic (e.g. MD-Logic) algorithms [8]-[9]. A recent development has been the bi-hormonal AP [10]-[11], which uses both insulin and glucagon. Comprehensive reviews of the latest advancements and current challenges in AP can be found in [12]-[15]. The increasing number of clinical trials has led to extensive in-hospital and, more recently, at-home evaluation of the feasibility of AP outside the controlled hospital environment. Most studies are restricted to the algorithmic evaluation of a patient cohort under uncertain conditions, such as erroneous meal intake and insulin sensitivity (SI) changes (e.g. physical activity).

In spite of these promising results, none of the currently proposed control strategies is intrinsically designed to handle uncertainties and personalisation. PID is designed for linear systems, MPC solves an open-loop optimisation problem which has proved sub-optimal in the presence of uncertainty [16] and MD-Logic is a rule-based approach directly subjected to the experience of the designer. In the view of patient variability, the algorithms have been enhanced with adaptive components, which are mainly based on the personalised identification of models involved [12] or correlation of algorithmic parameters with one or multiple patient-specific characteristics, such as body weight, correction factor or SI [10], [17], [18]. Nevertheless, the successful performance of the state-of-the-art AP algorithms proves that AP development is both feasible and viable and paves the path to a new era of more advanced algorithmic research towards robust and personalised insulin treatment.

Reinforcement learning (RL) is a branch of machine learning (ML) and is an intensively active research field which embraces algorithms that are able to learn from data and perform optimisation within uncertain environments. The field of RL falls between supervised and unsupervised learning and includes problems where an agent attempts to improve its performance at a given task over time by continual interaction with its environment [19]. RL began to develop as an independent branch in the early 1980s and was inspired by animal psychology and the idea of learning through trial-and-error. It was quickly adopted by the field of optimal control as a very efficient way to solve dynamic programming problems for which Bellman’s “curse of dimensionality” restricted an analytical solution. An extensive review of algorithms for RL has been presented in [20]. RL is field with an extensively investigated theoretical background, which is now finding its way towards practical application, due to modern advances in computational capacity [21]-[24]. In this view, the application in real life problems is highlighted as one of the current trends of RL. In medicine, RL is mainly investigated for prognosis, classification and diagnosis by means of big/heterogeneous data collection, fusion and analysis [25]-[29], with fewer reports on treatment studies [30], [31]. The advantages of ML and RL illustrate a promising path towards the resolution of the AP challenges, as has been recently recognised and reported [32], [33]. An online policy learning algorithm was presented in [34] and performed efficiently. To evaluate the algorithm, a deterministic gluco-regulatory model was used, but augmented with uncertainty to simulate patient variability. This process may not be representative of actual patient variability and limits the strength of the adaptive capability presented.

One factor that complicates the use of RL in medicine is the high number of constant and adaptive parameters which need to be tuned or initialised. Choosing the optimal values for these parameters is a challenging task and is usually performed manually, on the basis of problem-specific characteristics. However, in the face of inter-individual variability, this manual process may be unreliable or even unfeasible. Yet another criticism of RL is the difficulty in generalisation or qualitative explanation of both the learning process and the final solution (black box).

In the present study, an RL-based algorithm is proposed for personalised insulin infusion and glucose regulation in T1D. A model-free Actor-Critic (AC) algorithm is developed and evaluated *in silico* for its ability to maintain normoglycaemia within a large patient cohort and under variable environmental uncertainties. The scope of the study is two-fold: i) to investigate the applicability of RL in the context of a personalised AP and ii) to achieve an AC design that can be generalised and directly translated to medical experience. In order to overcome the tuning constraint discussed previously, the AC algorithm is enhanced with a novel method for automatic and personalised tuning, based on the estimation of information transfer (IT) from insulin to glucose signals.

Early-stage work in AC algorithms has already been presented, together with preliminary evaluation results [35], [36]. In the present study, the algorithmic and evaluation of the AC controller have been significantly improved. The control policy has been augmented by an exploratory policy, in order to increase the search space of the algorithm. Moreover, a supervisory control policy has been incorporated to enhance the algorithm’s safety. An important aspect of the current work is that the AC design is directly linked to physiological parameters and/or actions drawn from medical experience. In this approach, the automatic tuning method has been extended and associated to patient-specific characteristics. The estimation of IT has been further investigated in relation to the data used and the necessary data-length. For evaluation, the assessment of AC has been significantly extended to include multiple and more challenging protocols, with simultaneous meal uncertainty and diurnal SI variation.

In summary, the added value of this study on state-of-the-art algorithms for AP lies in the introduction of a novel control scheme able to meet the following challenges:

- Inter-/intra-patient variability and personalisation of insulin treatment through the use of a real-time adaptive learning algorithm
- Robustness from using a control algorithm which is suitable for optimisation under uncertainty
- Easy transfer to practice in hospital and at home since it is
  - based on limited a priori assumptions that counteract the high inter-patient variability
  - initialised on the basis of physiological parameters

The structure of this paper is as follows: Section 2 presents an analysis of AC algorithms. In Section 3, the design and development of AC for glucose regulation is presented, while the tuning of AC is discussed in Section 4. The results of the study are demonstrated in Section 5 and Section 6 summarises the final conclusions.

## The Actor-Critic Algorithm

The AC algorithm belongs to the class of RL and is characterised by the separation of the agent into two complementary parts: the Critic, responsible for evaluating the control policy and the Actor, responsible for improving control policy [37]. Within the RL family, the AC algorithms differ from actor-only or critic-only methods in that they possess better convergence properties. Moreover, their computational cost is lower, as they intrinsically estimate low variance gradients and parameterise policy to allow continuous time optimisation [38].

**Fig 1:**
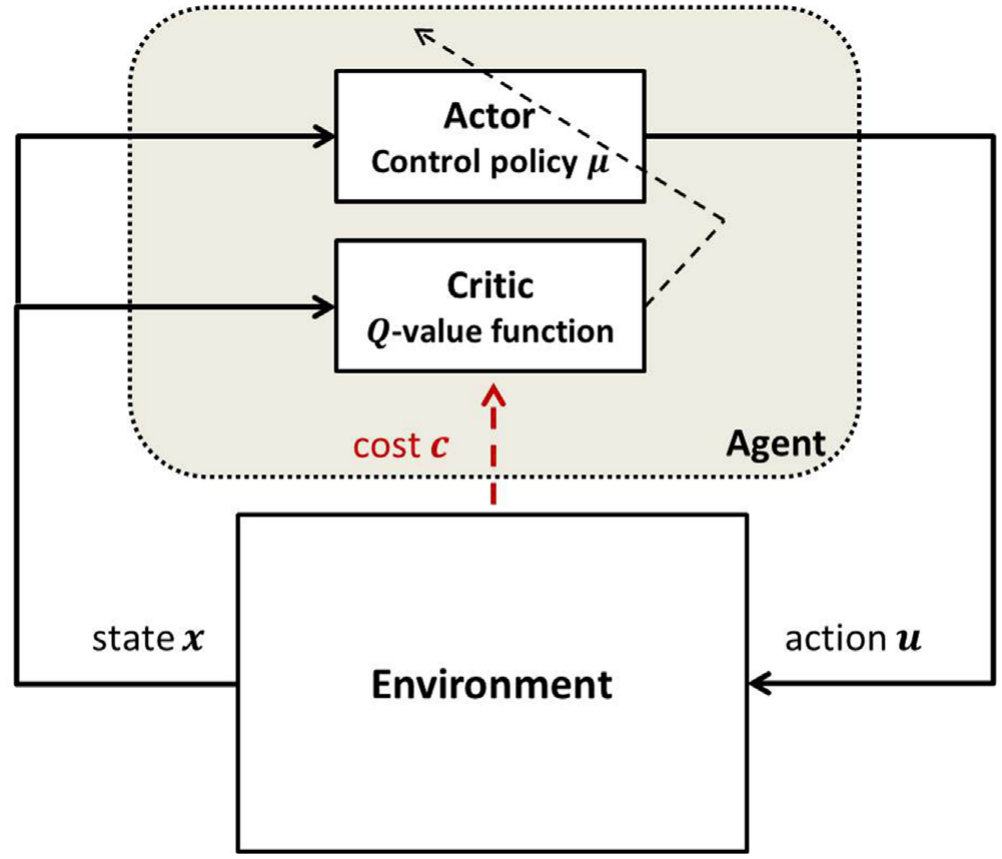
Schema of AC.

In AC learning, the agent follows a specific control policy and performs transitions between states within an uncertain environment. A schematic view of a system controlled by an AC algorithm is shown in Fig 1.

In the case of stochastic systems, the control policy is a conditional probability function *μ*(*u*|*x*,*θ*) from which control actions *u* are withdrawn, given current states of *x*. The aim of the agent is to find an optimal control policy, in order to minimise the expected cost throughout its path. Transition between states *x* and *y* depends on the chosen control action *u* and follows a transition probability distribution *p*(*y*|*x*,*u*). A local cost *c*(*x*,*u*) is associated with each state and action. In an average reward setting, the aim of the AC algorithm is to find an optimal control policy in order to minimise the average expected cost per state over all states. This is defined as:

$$\bar{a}(\theta )=\sum_{x\in X,u\in U}c(x,u)\eta_{\theta }(x,u)$$ (1)

where *η*_*θ*_(*x*,*u*) is the stationary probability of the Markov chain {*X*_*k*_,*U*_*k*_}.

### The Critic

The Critic agent is responsible for the evaluation of the current control policy on the basis of approximation of an associated expected cost. One of the most powerful methods used for this purpose is temporal difference (TD) learning [39], in which the total expected cost of a process that starts at state *x*, takes as first action *u* and follows policy *μ*(*u*|*x*,*θ*) that is defined through the value and action-value functions *V*_*θ*_(*x*) and *Q*_*θ*_(*x*,*u*), respectively:

$$V_{\theta }(x)=E[\sum_{k=0}^{\infty }\gamma^{k}c(x_{k},u_{k})|x_{0}=x]$$ (2)

$$Q_{\theta }(x,u)=E[\sum_{k=0}^{\infty }\gamma^{k}c(x_{k},u_{k})|x_{0}=x,u_{0}=u]$$ (3)

The value and action-value functions satisfy the following equations:

$$Q_{\theta }(x,u)=c(x,u)+\gamma \sum_{y}p(y|x,u)V_{\theta }(y)$$ (4)

$$V_{\theta }(x,u)=\sum_{u}\mu (u|x,\theta )\left[c(x,u)+\gamma \sum_{y}p(y|x,u)V_{\theta }(y)\right]\;\left(Bellman\;Equation\right)$$ (5)

For the given observed states, *x* = *x*_*k*−1_, *y* = *x*_*k*_ and action *u* = *u*_*k*−1_, and the Bellman Eq(5) reduces to:

$$V_{\theta }(x)=c(x,u)+\gamma V_{\theta }(y)$$ (6)

The Bellman’s curse of dimensionality restricts the analytical solution of Eq (6) in high dimensional spaces and requires the use of approximation methods. In the TD framework, the value function *V*(*x*) is approximated by a parameterised function *V*_*w*_(*x*) with *w*∈*R*^*K*^. The most commonly used architecture for the parameterised function is the linear approximation [40] defined as:

$${\tilde{V}}_{\theta }^{w}(x)=\sum_{i=1}^{K}w^{i}g_{\theta }^{i}(x)=w^{T}g_{\theta }(x)$$ (7)

where *g*_*θ*_(*x*) is a vector of basis functions of dimension *K*. Notation *w*^*T*^ denotes transpose. The approximation of the value function is performed via the estimation of the TD error *d* defined as the deviation of the approximated value function ${\tilde{V}}^{w}(x)$ from its subsequent estimation ${\tilde{V}}^{w}(y)$:

$$d=c(x,u)+\gamma {\tilde{V}}_{\theta }^{w}(y)-{\tilde{V}}_{\theta }^{w}(x)$$ (8)

On the basis of the TD error, the parameter vector *w* is updated according to the formula:

$$w_{k+1}=w_{k}+a_{k}d_{k}\sum_{n=0}^{k}\lambda^{k-n}\nabla_{w}{\tilde{V}}_{\theta }^{w}(x)=w_{k}+a_{k}d_{k}z_{k}$$ (9)

where *α*_*k*_ is a positive non-increasing learning rate sequence, 0 < *λ* < 1 is constant and *z*_*k*_ is the eligibility vector defined as:

$$z_{k}=\sum_{n=0}^{k}\lambda^{k-n}g_{\theta }(x_{n})$$ (10)

and are updated according to the following formula:

$$z_{k+1}=\lambda z_{k}+g_{\theta }(x_{k+1})$$ (11)

A similar process may be followed for the approximation of the action-value function *Q*_*θ*_(*x*,*u*):

$${\tilde{Q}}_{\theta }^{r}(x,u)=\sum_{i=1}^{L}r^{i}\varphi_{\theta }^{i}(x,u)=r^{T}\varphi_{\theta }(x,u)$$ (12)

where *φ*_*θ*_(*x*,*u*) is the vector of basis functions and *r* ∈ *R*^*L*^ is the respective parameter vector. A commonly used choice of the basis functions is *φ*_*θ*_(*x*,*u*) = *ψ*_*θ*_(*x*,*u*), where *ψ*_*θ*_(*x*,*u*) = ∇_*θ*_ ln *μ*(*u*|*x*,*θ*) is the likelihood ratio derivative of the control policy [37].

### The Actor

The aim of the Actor is to optimise the control policy over time towards minimisation of the average expected cost per state $\bar{\alpha }\left(\theta \right)$. Policy gradient methods are usually employed for the minimisation, which involve the estimation of the gradient $\nabla_{\theta }\bar{\alpha }\left(\theta \right)$ with respect to the policy parameter vector *θ*. The general policy update function has the following form:

$$\theta_{k+1}=\theta_{k}-\beta_{k}\nabla_{\theta }\bar{\alpha }(\theta )$$ (13)

where *β*_*k*_ is a positive sequence of learning rates. Various versions of Actor have been proposed, mainly distinguished by the approximation strategy for the gradient $\nabla_{\theta }\bar{\alpha }\left(\theta \right)$ [41]-[44]. In this study, the Actor update of [43] has been used in which:

$$\nabla_{\theta }\bar{\alpha }(\theta )=\sum_{x,y}\eta_{\theta }(x,u)d_{t}\psi_{\theta }(x,u)$$ (14)

## Glucose Regulation in T1D Based on an AC Algorithm

The AC algorithm is designed to optimise the insulin regime for each T1D patient. The insulin regime is defined as the combination of insulin basal rate (BR) and insulin:carbohydrate (IC) ratio. This choice was taken in order to be consistent with the medical practice; however, other insulin regime profiles may be used. The IC ratio is used for the calculation of the bolus dose (I_bolus_) according to the known carbohydrate (*CHO*) size of the upcoming meal as:

IC=IbolusCHO (15)

Prior to the design of the Critic and Actor agents, two important parameters of the algorithm need to be defined, i) the learning window, which corresponds to the update rate of the algorithm and ii) the state of the system. These are discussed in the following paragraphs.

### Learning window

The learning window is defined here as the period provided for data collection prior to an update of the insulin profile. There are several considerations that influence this decision. The learning window cannot be comparable to the loop delay introduced by the CGM and the sc insulin absorption. Moreover, the trade-off between fast and slow learning should be considered. Frequent updates may effectively follow the rapid glucose dynamics, but miss the “big picture” which carries more basic or generic information about the patient’s characteristics. Taking these into account, the optimisation window was chosen to be one day (24 hours). This choice also considers the 24-hour circle of the human body, which carries adequate information about the patient’s general glycaemic status. As a result, the insulin policy is evaluated and updated once per day as based on the respective daily glucose profile.

### System state

The dynamics of the glucoregulatory system are represented as a Markov decision process, where the state *x*_*k*_ is the status of the system in terms of hypo- and hyperglycaemia for day *k*. Define the glucose error *EG* at each time *t* as:

$$EG(t)=\{\begin{array}{c} G(t)-G_{h}\;if\;G≻G_{h}\\ G(t)-G_{l}\;if\;G≺G_{l}\\ 0\;else \end{array}$$ (16)

where *G*(*t*) is the glucose value at time *t* and *G*_*h*_ = 180*mg*/*dl*, *G*_*l*_ = 70*mg*/*dl* are the hyper- and hypoglycaemia bounds, respectively. The glycaemic profile of day *k* is described by two features related to the hyper- and hypoglycaemic status of that day and more specifically to the average daily hypoglycaemia and hyperglycaemia error:

$$x_{k}^{1}=\frac{1}{N_{1}}\sum_{t\;\in \;day\;k}H\left(EG(t)\right)$$ (17a)

$$x_{k}^{2}=\frac{1}{N_{2}}\sum_{t\;\in \;day\;k}H\left(-EG(t)\right)$$ (17b)

where *H*(⋅) is the Heaviside function and *N*_*i*_ is the number of time samples above the hyperglycaemia (*i* = 1) or below the hypoglycaemia (*i* = 2) threshold. Firstly, the features are normalised in [0 1]. The normalised features formulate the state $x_{k}={\left[x_{k}^{1}\;x_{k}^{2}\right]}^{T}$ of day *k*.

### Design of the Critic

The mathematical formulation of the Critic was given in Section 2. At the end of day *k*, the glucose profile of the day is collected and the state *x*_*k*_ is calculated. On the basis of the state, a local cost *c*(*x*_*k*_) is assigned, defined as:

$$c(x_{k})=a_{h}x_{k}^{1}+a_{l}x_{k}^{2}$$ (18)

The weights *a*_*h*_ and *a*_*l*_ are used for scaling the hypo- and hyperglycaemia components and are chosen as *a*_*h*_ = 1 and *a*_*l*_ = 10 [35]. The action-value function is linearly approximated as described in Eq (12). The basis functions *φ*(⋅) are set equal to the likelihood ratio derivative (LRD) [37] of the control policy which will be derived in a later phase. For the Critic update, the constants *γ* and *λ* are chosen as *γ* = 0.9 and *λ* = 0.5 for all patients. The Critic’s learning rate is set $a_{k}^{c}=0.5$ for all patients. These values were found experimentally. The initial parameters *r*_0_ are set to random values in [–1 1] and the initial parameters *z*_0_ to zero values for all patients.

### Design of the Actor

The Actor implements a dual stochastic control policy *μ*(*u*_*k*_|*x*_*k*_,*θ*_*k*_) for the daily optimisation of the BR and IC ratio starting from an initial BR (IC ratio) value. In order to dissociate the action from the absolute level of the current insulin regime, the control action *u*_*k*_ is defined as the rate of change of BR (IC ratio) from day *k*-1 to day *k*. The benefit of this choice will be revealed later. Thus, the BR (IC ratio) is updated as follows:

$$S_{k}=S_{k-1}+P_{k}^{S}S_{k-1}$$ (19)

$$P_{k}^{S}=u_{k}^{S}\sim \mu (u_{k}^{S}|x_{k},\theta_{k}^{S})$$ (20)

where *S* = {BR, IC} and $P_{k}^{S}$ is the control action i.e. the rate of change of *S*_*k*_ from day *k*-1 to day *k*. The final applied control action $P_{k}^{S}$ is withdrawn from the probability distribution $\mu (u_{k}^{S}|x_{k},\theta_{k}^{S})$ of the control policy based on the current state *x*_*k*_ and policy parameter vector $\theta_{k}^{S}$. For the design of the probability distribution, a three-step process is followed, based on the generation of three different types of control actions: i) linear deterministic, ii) supervisory and iii) exploratory action. Hereafter, the notations *k* and *S* are omitted for clarity purposes. The procedure is exactly the same for BR and the IC ratio.

The linear deterministic control action *P*_*a*_ is defined as the linear combination of the current state and policy parameter vector:

$$P_{a}=x^{T}\theta$$ (21)

In other words, this control action associates the daily hypo- and hyperglycaemic status to the needed percentage of BR (IC ratio) change for the next day.

The supervisory control action *P*_*s*_ is a conservative rule-based advice to the algorithm and mainly serves as guidance of the direction of change to be followed and as a safety module against extreme insulin changes by the algorithm [45]. The supervisory action is defined as:

$$P_{s}=\{\begin{array}{l} 0\;if\;x_{1}=x_{2}=0\\ \pm 0.1x_{1}\;if\;x_{1}≻0\;and\;x_{2}=0\\ \mp 0.1x_{2}\;if\;x_{2}≻0\; \end{array}$$ (22)

where the upper sign refers to BR and the lower sign to IC ratio.

The weighted sum of the two previous actions defines the total deterministic control action *P*_*d*_:

$$P_{d}=hP_{a}+(1-h)P_{s}$$ (23)

where *h* is a factor that allows us to scale the contribution of each part to the final output. In this study, the weighting factor has been chosen as *h* = 0.5 and thus assigns equal contributions to the two actions.

The exploratory control action *P*_*e*_ occurs by adding white noise to the final deterministic policy as below:

$$P_{e}=P_{d}+N(0,\sigma )$$ (24)

where *N*(0, *σ*) is white Gaussian noise with zero mean and standard deviation *σ*. The aim of the exploration process is to widen the search space of the algorithm in order to optimise the performance and the convergence rate. The result of the exploration process is the final control action to be applied.

Based on the previous analysis, we are now ready to derive the control policy *μ*(*u*|*x*,*θ*) as the probability distribution from which the final control action *u* = *P*_*e*_ is withdrawn:

$$\mu (u|x,\theta )=\frac{1}{\sigma \sqrt{2\pi }}\text{exp}\left(-\frac{1}{2}{\left(\frac{u-P_{d}(x)}{\sigma }\right)}^{2}\right)$$ (25)

The control policy is a Gaussian probability distribution with mean equal to the total deterministic action *P*_*d*_(*x*) and standard deviation *σ*. Finally, the LRD *ψ*_*θ*_(*x*,*u*) has to be derived. Taking the gradient of the control policy with respect to *θ* we have:

$$\nabla_{\theta }\mu (u|x,\theta )=\mu (u|x,\theta )\frac{u-P_{d}(x)}{\sigma^{2}}\nabla_{\theta }P_{d}(x)$$ (26)

From Eqs (25) and (26), LRD becomes:

$$\psi_{\theta }(x,u)=\nabla_{\theta }\text{ln}\mu (u|x,\theta )=\frac{u-P_{d}(x)}{\sigma^{2}}\nabla_{\theta }P_{d}(x)$$ (27)

and the policy parameter update of the Actor is defined as follows:

$$\theta_{k+1}=\theta_{k}-\beta_{k}d_{k}\psi_{\theta_{k}}(x_{k},\mu_{\theta_{k}}(u_{k}|x_{k}))=\theta_{k}-\beta_{k}d_{k}\frac{u-P_{d}(x)}{\sigma^{2}}\nabla_{\theta }P_{d}(x)$$ (28)

It can be seen in Eq (28) that the update of the policy parameter vector depends on the difference between the total deterministic and the exploratory policy, i.e. on the noise variance *σ*^2^. When an optimal policy has been found, which results in a state *x*_*k*_ ~0, we would like to reduce the exploration, as this may lead the system away from the solution found. To this end, the variance *σ*^2^ is defined as a function of the state *x*_*k*_:

$$\sigma^{2}=KS\left\|x_{k}{}^{2}\right\|$$ (29)

The larger the state *x*_*k*_, the greater the time spent in hypo-/hyperglycaemia on day *k*, i.e. the larger the exploration space for a better control policy. The constant *KS* is set manually to 0.05 following a trial-and-error process. The Actor learning rate *β*_*k*_ is set equal to the variance *σ*^2^ using the same reasoning. In this way, the AC algorithm is all-time learning, in order to compensate for temporal or permanent changes in the gluco-regulatory system of each patient.

## Personalised Tuning of the AC Algorithm

The design of the AC algorithm, as described in the previous section, involves various parameters that need to be tuned. Taking into account the patient variability, personalised tuning might be required for some of the parameters. Manual tuning for each patient is infeasible or might compromise the patients’ safety, so automatic methods need to be investigated.

On the basis of preliminary simulations and under different tuning configurations, the AC parameters were first split into two classes, as robust (R) or sensitive (S). The parameters included in the R class were associated with low sensitivity to patient variability and were manually tuned by empirical methods, with common values for all patients given in the previous section. The S class included the parameters which were found to be sensitive to patient-specific characteristics. The parameters identified in this class were the initial values of the BR and IC ratio and the Actor’s initial policy parameter vector *θ*_0_. For the first two parameters, universal tuning is not possible, as the insulin requirements naturally differ between different diabetic patients. It will be shown that the policy parameter vector *θ* is tightly related to patient-specific characteristics and its initial tuning affects both the performance and convergence rate of the algorithm. Thus, automatic, individualised tuning procedures were followed for the S class parameters. Table 1 summarizes the parameters of the AC algorithm along with their description, values and tuning class.

**Table 1:** Parameters of the AC algorithm.

Parameter	Description	Value	Class
*a*_*h*_	Local cost hyperglycemia weight	1	R
*a*_*l*_	Local cost hyporglycemia weight	1	R
*γ*	Discount factor long-term cost	0.9	R
*λ*	TD learning constant	0.5	R
$a_{k}^{C}$	Critic learning rate	0.5	R
$a_{k}^{A}$	Actor learning rate	1	R
*r*_0_	Critic initial parameter vector	Random in [–1 1]	R
*z*_0_	Critic initial eligibility vector	Zero	R
*σ*	Standard deviation exploration action	0.05	R
*S*_0_	Actor initial BR/IC ratio	patient-specific	S
*θ*_0_	Actor initial parameter vector	patient-specific	S

### Initialisation of BR and IC ratio

In order to guarantee safety, the initial values for the BR and IC ratio should be specific and appropriate for each patient. Clinical experience in treating diabetes has developed a number of empirical rules for the estimation of BR profiles and IC ratios for patients under CSII pump therapy, as based on their body weight, SI and lifestyle factors [46]. These rules provide an open-loop insulin regime which may not be optimal but ensures primary glucose regulation. Thus, when applied in clinical practice, the BR and IC ratio of the AC algorithm can be initialised using the patient’s individual values as optimised by his/her physician. This practice has the additional advantage that the transition of a patient from CSII to AP can be smoother both for him/herself and the physician.

### Initialisation of policy parameter vector *θ*

Initialisation of the policy parameter vector *θ* was based on investigation of its natural representation within the designed insulin infusion control algorithm. The optimal values of the policy parameter vector *θ* answers the question: “How much should we change BR and IC ratio based on the observed daily hyper-/hypoglycaemia?” The answer is directly related to the patient’s SI and depends on his/her body mass index (BMI), total daily insulin (TDI) needs, lifestyle and genetic factors. Estimation of SI is currently performed in a clinical environment using clamp or intravenous glucose tolerance tests, which are time consuming and costly. In recent years, there have been efforts to achieve online estimation of SI to be incorporated into AP algorithms, using CGM and insulin pump data and based on the inverse solution of a diabetes physiological model [12], [47].

Often in practice, SI is directly related to a patient’s TDI, as this information is easily accessible. However, even for two patients with the same TDI and BMI, the impact of 1 U of insulin may be different. In this study, we capture this difference through the IT from insulin to glucose signals. The insulin-to-glucose IT was measured using the notion of transfer entropy (TE), a very powerful method for the estimation of IT in non-linear random processes [48]. TE estimates the IT from a cause signal *Υ* (insulin) to an effect signal *X* (glucose). This value is independent of the magnitude of the two signals, i.e. the amount of insulin and the glucose concentration. For two patients with the same TE, higher TDI corresponds to lower SI. Similarly, if two patients have the same TDI, higher TE can be translated to lower SI. Following this reasoning, information about a patient’s SI was estimated as:

$$\tilde{SI}=c_{1}\frac{\text{TE}}{\text{TDI}}$$ (30)

where *c*_1_ is a positive constant. Given the definition of SI, if a patient wants to reduce his/her glucose levels by Δ*G*, the necessary amount of insulin should be:

$$I_{\Delta G}=\frac{\Delta G}{\text{SI}}$$ (31)

Substituting SI with its estimation $\tilde{SI}$ given in Eq (30), we have:

$$I_{\Delta G}=c\frac{\Delta G}{\text{TE}}\text{TDI}$$ (32)

where *c* = 1/*c*_1_. In the case of the AC algorithm, the aim is to find the optimal change in the BR and IC ratio in order to eliminate daily hypo- and hyperglycaemia. This can be seen as a parallel to Eq (32):

$$\Delta S^{i}=c\prime \frac{x^{i}}{\text{TE}}\text{TDI}$$ (33)

where *x*_*i*_ is the hyperglycaemia (*i =* 1) or hypoglycaemia (*i =* 2) feature, i.e. the average daily hypo-/ hyperglycaemic error as defined in (29a, b), Δ*S*^*i*^ is the change in BR or IC ratio based on the respective feature and c' a positive constant. Considering that TDI is directly reflected in the daily BR and IC ratio, Eq (33) can be rewritten as:

$$\Delta S^{i}=c\prime \frac{x^{i}}{\text{TE}}S$$ (34)

If we set $\theta^{i}=\frac{c\prime }{\text{TE}}$, Eq (34) becomes:

$$\Delta S^{i}=\theta^{i}x^{i}S$$ (35)

and the total change in BR or IC ratio based on both hypo- and hyperglycaemia features is the linear combination of their respective contributions as:

$$\Delta S=\Delta S^{1}+\Delta S^{2}=\theta^{1}x^{2}S+\theta^{2}x^{2}S=(\theta^{1}x^{1}+\theta^{2}x^{2})S=\theta^{T}xS$$ (36)

where *θ* = [*θ*_1_ *θ*_2_]^T^ and *x* = [*x*_1_ *x*_2_]^T^ is the feature vector. Finally, if we set *P*_*s*_ = *θ*^T^ *x* then Eq (35) becomes:

$$\Delta S=P^{S}S$$ (37)

where *P*^*S*^ is the percentage of change of *S* and represents AC deterministic control action as previously defined in Eq (33).

The aforementioned analysis illustrates that defining the control action as the rate of insulin change permitted tuning of AC, using the insulin to glucose IT and without the need to estimate SI, which would be a more cumbersome process. The analysis is approximate and may only be used as a draft estimate of the necessary BR or IC update. However, the scope is to provide a better starting point to AC in order to enhance the optimisation process. The initial values of the policy parameter vector for patient *p* are set as:

$$\theta_{0}^{S}(p)=\left[\frac{W_{h}}{TE(p)}\;\frac{W_{l}}{TE(p)}\right]$$ (38)

where *W*_*h*_ and *W*_*l*_ are weights related to the hyper- and hypoglycaemia features, respectively, set manually as *W*_*h*_ = 0.1 and *W*_*l*_ = −0.2 for all patients. Again, a higher value is assigned to the hypoglycaemic weight, as avoiding hypoglycaemia has higher priority.

### Estimation of insulin-to-glucose TE

**Fig 2:**
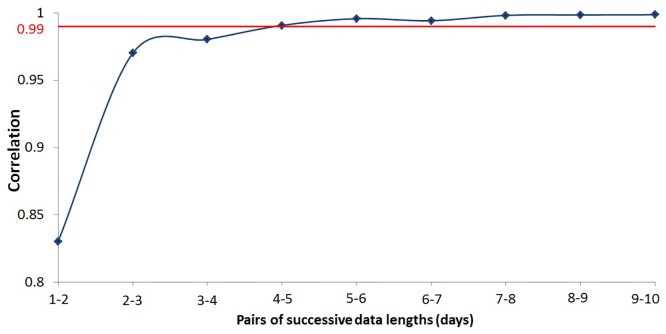
Correlation of TE values for successive pairs of data lengths.

Insulin-to-glucose TE is estimated on the basis of CGM and insulin pump data for four days collected from each patient. In order to choose the appropriate data size, datasets of different durations were used and the correlation between the respective TE values was computed for successive data lengths (Fig 2). It was observed that data of four days or more gave highly correlated TE values (>99%).

The estimation of TE was based on the following formula:

$$TE_{IA\to G}=\sum_{t}p(G_{t},G_{t-1},IA_{t-d})\text{log}\frac{p(G_{t}|G_{t-1},IA_{t-d})}{p(G_{t}|G_{t-1})}$$ (39)

where *G*_*t*_, *IA*_*t*_ are the glucose and active insulin at time *t* and *d* is the insulin time-delay set here as *d* = 20 minutes, according to the average physiological insulin absorption delay for rapid-acting insulin analogues. Active insulin was estimated as the sum of insulin on board (*IOB*) related to the bolus doses and basal insulin infusion:

$$IA(t)=IOB(t)+I_{basal}(t)$$ (40)

Estimation of *IOB* was based on [49]. For the estimation of the probability distributions, the fixed data partitioning method was used, in which the time-series are partitioned into equal sized bins and the probability distributions are approximated as histograms [50]. The size of the partition bins for glucose and insulin was chosen as *G*_*bin*_ = 10 mg/dl and *IA*_*bin*_ = 1 U, respectively.

## Results and Discussion

The AC algorithm was evaluated *in silico* in a series of experiments designed in order to capture realistic conditions of every-day living with T1D. The evaluation criteria were the time spent in normoglycaemia (70≤*G*≤180 mg/dl), mild hypoglycaemia (50≤*G*<70 mg/dl), severe hypoglycaemia (*G*<50 mg/dl), mild hyperglycaemia (180<*G*≤300 mg/dl) and severe hyperglycaemia (*G*>300 mg/dl) as well as the Low Blood Glucose Index (LBGI) defined in [51].

### Experimental protocol

#### Simulation environment

In all experiments, the FDA accepted UVA/Padova T1DM simulator was used [52], [53]. All experiments were first tested on the 30-patient cohort of the educational version. Of the 30 patients, 2 children presented excessive glucose fluctuations and were excluded as outliers. Similar observations have been reported by other teams [54]. In order to enhance the validity of the results, the most representative experiments were subsequently tested on the 100 FDA accepted adult population of the full version of the simulator. The simulator provides optimised BR and IC ratio values which can be assumed to be the standard treatment of the patients defined by their physician.

#### Meal protocol

The meal protocol used is described in detail in [55]. The meals were announced to the controller 30 minutes prior to intake. In order to simulate the common errors of diabetic patients in CHO counting, a random meal uncertainty was introduced in all experiments; this was uniformly distributed between -50% and +50%.

#### SI variation

A scenario of varying SI was designed in order to simulate physiological diurnal (intra-day) SI variations. Two cases of SI variation were simulated, i) the dawn phenomenon and ii) physical activity. More specifically, every day of the trial, SI drops between 04:00 and 08:00 to -25% of its nominal value. SI ramps up or down within a time-frame of 30 minutes. Furthermore, three days per week, the patients perform physical activity between 18:00 and 20:00, which results in an increase in SI up to 33% of its nominal value. SI ramps up within 30 minutes and ramps back down at the end of the physical activity within four hours.

### Experiments

A total of six experiments were conducted as described below. The same meal protocol was used in all experiments.

*E1*: The optimised BR and IC ratio provided by the simulator were applied fixed in an open-loop (OL) approach simulating standard treatment. SI was steady throughout the trial with no variations. The trial lasted four days.

*E2*: The same as E1 including SI variation.

*E3*: The AC algorithm was applied without the automatic TE-based tuning. The initial parameter vector *θ*_0_ as set to zero values for all patients. SI was steady throughout the trial with no variations. The trial lasted 14 days. The first four days OL glucose control was applied as in E1. Closed loop (CL) control with AC started on day 5. The next first five days were considered as the training phase of the AC algorithm and the rest were used for evaluation.

*E4*: The same as E3 including SI variation

*E5*: The same as E3 but the AC policy parameter vector *θ*_0_ was individually initialised based on the TE approach.

*E6*: The same as E5 including SI variation.

All experiments were tested on the educational version of the UVA/Padova T1DM simulator, while experiments E5 and E6 were further tested on the full version as well.

### 28-subject cohort

The performance of the AC algorithm is presented in Table 2 for the three age groups of patients and all experiments. The results of E3-E6 refer to the last five days (evaluation period) of the closed loop session. The CL insulin infusion results in improved glycaemic control for all patients, especially in a reduction in the time spent in the hypoglycaemic range while preserving an extensive period in the target range.

**Table 2:** Percentage of time spent in the target range, mild hypoglycaemia, severe hypoglycaemia, mild hyperglycaemia and severe hyperglycaemia for each age group and the six experiments.

Glucose Levels	E1	E2	E3	E4	E5	E6
*Adults*						
70–180 mg/dl	97.18	94.43	96.92	96.30	96.28	94.96
50–70 mg/dl	1.47	2.18	0.31	0.20	0.16	0.09
< 50 mg/dl	0.31	1.04	0.00	0.00	0.00	0.00
180–300 mg/dl	1.03	2.35	2.76	3.50	3.56	4.96
> 300 mg/dl	0.00	0.00	0.00	0.00	0.00	0.00
*Adolescents*						
70–180 mg/dl	86.44	82.73	81.72	79.59	81.64	77.81
50–70 mg/dl	2.39	3.23	0.75	0.98	0.77	1.38
< 50 mg/dl	0.01	1.64	0.00	0.01	0.00	0.05
180–300 mg/dl	11.07	12.30	17.08	19.13	17.12	20.55
> 300 mg/dl	0.10	0.10	0.45	0.29	0.47	0.21
*Children*						
70–180 mg/dl	74.77	75.82	79.30	80.52	79.24	77.36
50–70 mg/dl	14.63	12.21	2.19	2.72	1.27	1.35
< 50 mg/dl	6.15	7.33	0.20	0.33	0.06	0.05
180–300 mg/dl	4.37	4.56	16.74	16.17	18.81	20.86
> 300 mg/dl	0.08	0.08	1.58	0.27	0.61	0.38

Compared to OL (E1, E2), AC reduced the time spent in mild hypoglycaemia by at least 79% in adults, 62% in adolescents and 78% in children while its contribution in severe hypoglycaemia was even higher, with 100% reduction in adults, over 99% in adolescents and 96% in children. The contribution of AC is mostly significant in children who, during OL, presented unacceptably long periods spent in mild and severe hypoglycaemia. Even in the presence of SI variation, where the OL control could not prevent incidents of severe hypoglycaemia, AC was able to reduce the hypoglycaemic events and maintain very long periods spent in the target range.

**Fig 3:**
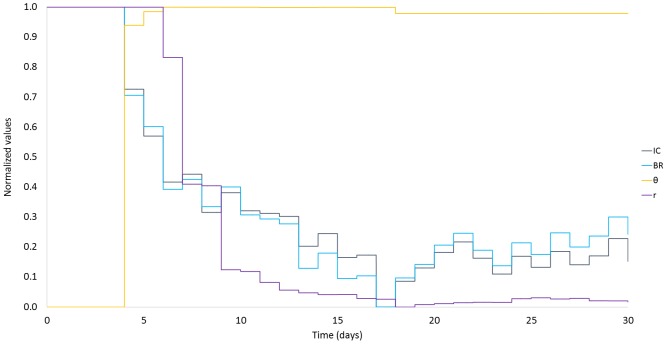
Evolution of the AC adaptive parameters for one *in silico* child under an extended E6 for 30 days.

Through these results, it is important, also, to investigate the internal performance of the AC algorithm. Fig 3 illustrates the evolution of the AC adaptive parameters for one *in silico* child during experiment E6. The child starts with insulin regime higher than required resulting in long hypoglycemic events. The AC parameters are gradually adapted leading to the reduction of BR and IC ratio and the efficient regulation of the glucose profile. For clarity only one of Critic’s parameters *r* and one of Actor’s parameters *θ* is shown along with the BR and the IC ratio, all normalized in [0, 1]. In order to demonstrate the convergence of the AC, the E6 experiment was extended to 30 days. From Fig 3 can be seen that after the 14^th^ day the parameters remain mostly stable.

#### Manual vs. automatic AC tuning

From Table 2, it can be seen that the automatic TE-based AC (E5, E6) resulted in lower time spent in mild and severe hypoglycaemia for adults and children, while for adolescents the manual-based AC seems to perform better in these terms. Performance of a Student’s t test showed that manual and TE-based initialisation in hypoglycaemia prevention (in terms of time spent in this region and LBGI) were statistically different in both adults and children (p values<0.05), but not in adolescents. In all cases, though, the absolute differences between the two methods were small.

**Fig 4:**
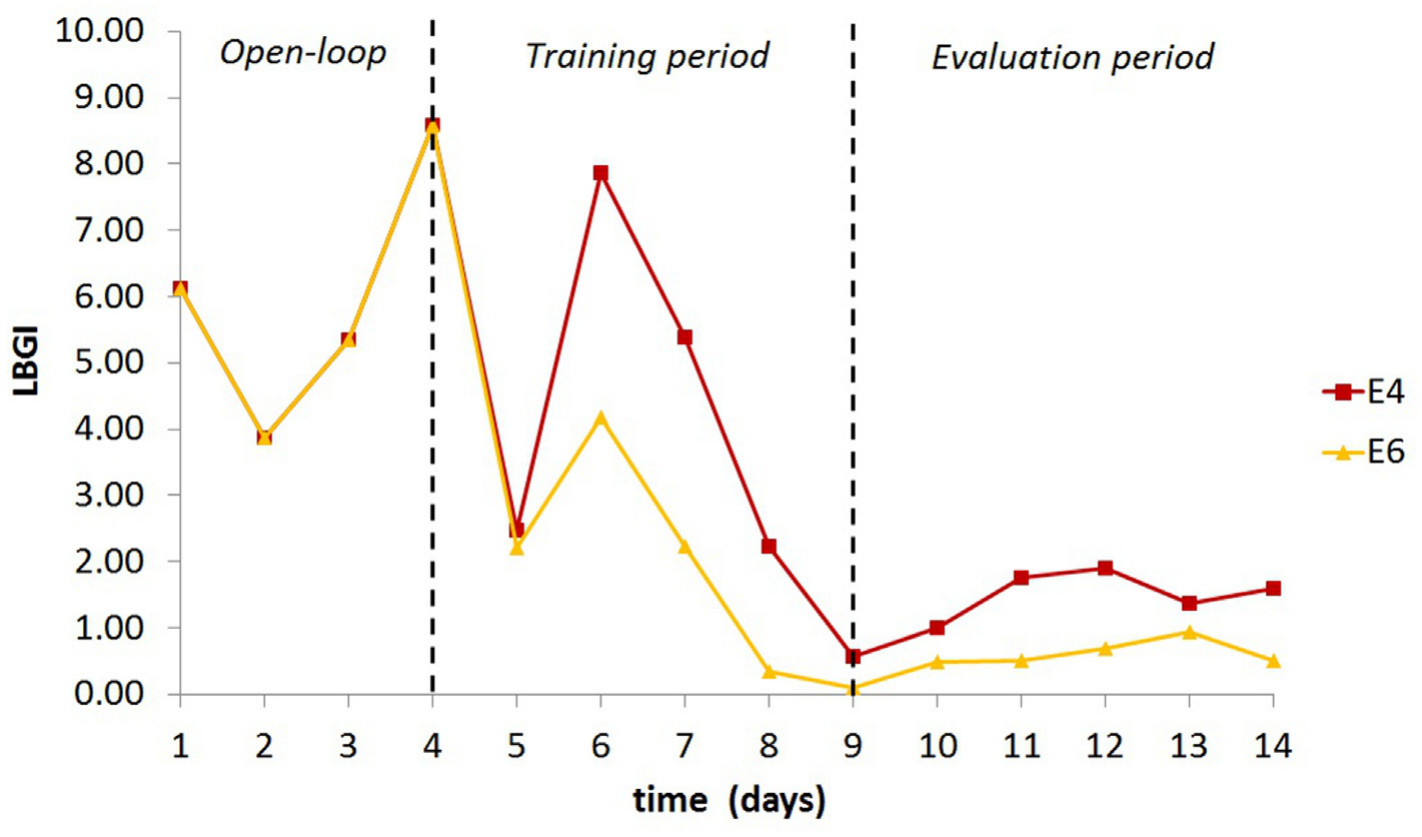
LBGI progress of one *in silico* child during experiments E4 and E6.

It is important to mention that the contribution of automatic initialisation may not be of equal importance for all patients. Patients with high TE, which (as discussed earlier) can be attributed to an aspect of SI, did not show significant improvement with automatic initialisation. These patients are expected to need small insulin updates (meaning here the percentage of change from the current insulin BR or IC ratio); thus the initial AC parameter vector *θ*_0_ will be close to zero. However, for patients with lower TE, the contribution was important during both the training and the evaluation period. This is not a surprise, given that patients with low TE will require larger updates of their insulin regime in order to improve their glucose profile. An example of such a case is illustrated in Fig 4, where the LBGI progress of one *in silico* child with TE below the average is presented for E4 and E6.

#### SI variation

When OL control is applied, introduction of SI variation resulted in increased time spent in both mild and severe hypoglycaemia. The effect of SI variation is mostly significant in adolescents, but it is observable in all age groups. However, during closed loop glucose control based on the AC algorithm, no significant difference is observed between E3-E4 and E5-E6, as shown in Table 2. This fact reveals that AC is robust against system uncertainties, and is able to account for them and optimise its performance respectively.

### 100-adult cohort

The AC algorithm was further evaluated using the 100 FDA-accepted adult population with the UVA/Padova T1DM simulator. The purpose of this evaluation was two-fold. On the one hand, it enhances the validity of the results, due to the use of a very large patient database. On the other hand, it offers the chance to comparatively assess the performance of AC against state-of-the-art glucose control algorithms which have been evaluated using the same simulator and patient database. The performance of AC for the 100-adult cohort during experiments E5 and E6 is presented in Table 3.

**Table 3:** Percentage of time spent in target range, hypoglycaemia and hyperglycaemia for AC evaluated in the 100-adult cohort under E5 and E6.

Glucose Levels	E5	E6
70–180 mg/dl	95.66	93.02
< 70 mg/dl	0.27	0.27
> 180 mg/dl	4.07	6.71

**Fig 5:**
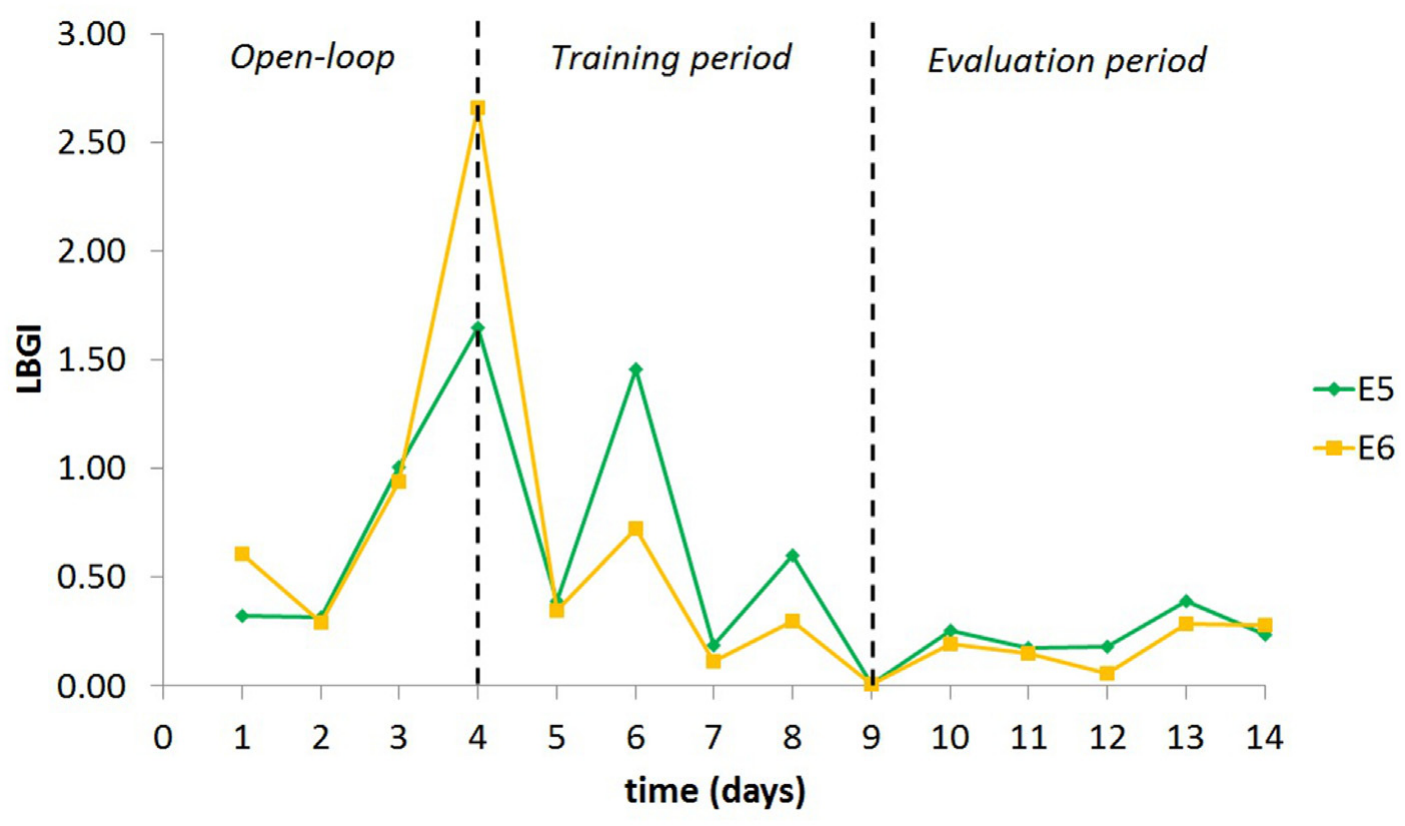
Daily LBGI of the total experiment duration for the 100 adult patients and experiments E5-E6.

When SI variation was introduced (E6), two of the 100 adult patients exhibited problematic performance. Both patients reached glucose levels below 40 mg/dl during the open loop period. It was not possible for the AC algorithm to bring these patients back during closed loop and certainly this is beyond the scope of any glucose control algorithm. As would have been the case in a real clinical study, these two patients have been excluded from the evaluation. Thus, the results of E6 refer to 98 adults. From Table 3, it can be seen that the AC algorithm performs excellently with very long periods spent in the target range and very few hypo- and hyperglycaemic events. During OL (first 4 days), introduction of SI variation increased the time spent in mild hypoglycaemia by 44% and in severe hypoglycaemia by 770%. During CL with the AC algorithm, the time spent in hypoglycaemia was the same in E5 and E6 and was preserved at very low levels in both cases. This can be further illustrated in Fig 5, which presents the daily LBGI progress for the whole duration of E5 and E6. These results support the previously discussed performance of the AC algorithm based on the training version of the simulator. They further demonstrate that AC can provide personalised insulin treatment and achieve tight glucose regulation even under high patient variability and other uncertainties.

The results of this study demonstrate that adaptive control methods can be a valuable tool in personalization and optimization of insulin treatment in T1D. Moreover, the study illustrates the challenges in the application of such algorithmic approaches and proposes strategies to address them. It is, however, very important to note that the role of the physician is not supressed by an AP system, not even if the AP has self-learning capabilities. In the development of AP algorithms it is important to take this fact into account and leave room for the vivid interaction of the system with the physician [56].

## Conclusions

An AC learning algorithm, chosen from the family of RL, was proposed for the design and development of a personalised AP in T1D. The AC algorithm was evaluated *in silico* using the FDA-accepted T1D simulator under a complex meal protocol and diurnal SI variation. The results of the study illustrate that AC was able to learn in real-time patient-specific characteristics captured in the daily glucose profile and provide individualised insulin treatment. The algorithm achieved very high time spent in the target range, with effective limitation of hypoglycaemia under uncertainty in the CHO content and diurnal SI. The novel automatic and personalised tuning method contributed in the optimisation of the algorithm’s performance. Compared to other *in silico* tested control strategies for glucose regulation, AC was evaluated under more complex experimental protocol and presented comparable or superior results. However, due to the differences in the evaluation scenarios, a direct comparison with solid conclusions cannot be performed.

The AC design took into account the characteristics of medical practice in an effort to present a comprehensive and easily adaptable structure. The feasibility of personalised tuning through link to physiological parameters was illustrated. This fact releases the applicability of RL algorithms in T1D from a very important constraint.

It is worth noting that the design of the AC algorithm permits its direct use by the patient without initial clinical preparation by the physician as it is self-adaptive and relies on the patient’s current standard treatment as a starting operation point. During the first four operation days, AC provides the patient’s standard treatment as defined by his/her physician and, in parallel, collects his/her CGM and insulin pump data. At the end of this period, the algorithm automatically estimates the TE and initialises the policy parameters. In sequence, AC continues the personalisation of insulin treatment with daily adaptation of BR and IC ratio. For all involved calculations, AC needs minimal computational time and can run smoothly on a mobile device.

In the present configuration, the AC algorithm is designed to follow and learn the slow glucose dynamics captured in the daily glucose profile. Different learning configurations may be investigated with shorter update windows and different cost functions, bearing in mind the trade-off between fast and slow learning. Alternatively, AC could be combined with existing control strategies, independently of the used algorithm, which provide short-term insulin updates, in order to build a control system able to capture both the fast and the slow glucose dynamics. Moreover, AC could have an additional medical impact as a personalised advisory system for the physicians.

The aforementioned potentialities of AC will be investigated in the near future. The next steps also include the extensive investigation of insulin to glucose IT and its correlation with SI and TDI. Alternative patient-specific characteristics will be examined for the automatic AC initialisation. Moreover, the AC algorithm will be enhanced with additional systems for the estimation of the precise CHO content of meals [57] as well as physical activity. As soon as the final algorithmic version is established, extensive clinical evaluation will follow, both at hospital and at home, according to the evaluation guidelines defined by FDA.
